# Consumer Perceptions of Oxidation‐Related Effects on Facial Skin Aging and Skincare Product Features

**DOI:** 10.1111/jocd.70623

**Published:** 2025-12-19

**Authors:** Kelly Chua, Sharon Shi, Bee Leng Lua

**Affiliations:** ^1^ Groupe Clarins Singapore Singapore

**Keywords:** consumer perception, facial skincare, online survey, oxidation‐related skin aging effects, self‐administered questionnaire

## Abstract

**Background:**

Oxidative stress on the skin can exacerbate undesirable skin conditions and promote skin aging. It is unclear how consumers of skincare products recognize, perceive, and describe oxidation effects in skin. It is also unclear what they perceive to be the solution for oxidation in the skin.

**Aims:**

To determine which facial skin qualities consumers associate with oxidation, and to determine skincare product qualities that consumers associate with anti‐oxidation.

**Methods:**

Focus group discussions (FGDs) with 24 female volunteers (aged 25–50 years), and an online survey with 800 female volunteers (aged 20–50 years), were conducted across four Chinese cities (Shanghai, Beijing, Chengdu, and Xi'an) in September 2024. Females of all skin types who regularly used skincare products were eligible to participate. Total unduplicated reach and frequency (TURF) and principal component analyses (PCA) were performed on data collected from the online surveys.

**Results:**

Consumers mainly associated oxidation‐related skin effects with the skincare descriptive categories “repair,” “firming,” and “skin and spot lightening.” Consumers who had skin oxidation concerns made a stronger association with “skin and spot lightening” than those who did not have oxidation concerns. TURF analysis found skincare products with the descriptive categories “repair” and “skin and spot lightening” can reach 73% of the surveyed female skincare consumer population.

**Conclusion:**

Participants associated antioxidant effects with facial skin repair and spot lightening. These associations are stronger for participants who have skin aging concerns. Skincare products that can promote skin repair and lightening are estimated to reach 73% of the female skincare consumers similar to the study participants.

## 1 Introduction

It is currently believed that cellular oxidative stress (i.e., free radical formation) is one of the factors contributing to skin aging in humans [1]. The main mechanism underlying oxidative stress‐induced skin damage is the generation of reactive oxygen species (ROS) [2]. ROS accumulate over time in skin cells. The ROS load in the skin is higher than in any other organ, and has been correlated with skin aging [1]. ROS accumulation in the skin can come from both external insults (e.g., solar ultraviolet, infrared and visible light, environmental pollution) and internal insults (e.g., psychological stress, poor diet) [3].

Oxidative stress on the skin can lead to skin aging by exacerbating undesirable skin conditions such as impaired skin barrier, skin tone unevenness, pigmentary disorder, skin roughness and wrinkles [1, 3]. The cellular mechanisms underlying this effect are the ROS‐induced degradation of cellular building blocks including DNA, proteins, and lipids. ROS also alters key signaling pathways, which can alter cytokine release and enzyme expression [2].

To combat the skin aging effects of oxidation, the skincare industry has developed skincare incorporating naturally derived and synthetic antioxidants, which act to neutralize free radicals to help prevent cellular damage. Several antioxidants, such as vitamin C, ferulic acid, resveratrol, have been shown to reduce the signs of skin aging and pigmentation [3].

It is unclear how consumers of skincare products recognize, perceive, and describe oxidation effects in skin. It is also unclear what they perceive to be the solution for oxidation in the skin. Hence, to better understand consumer perceptions of oxidation on the skin, this study employed qualitative focus group discussions and an online survey to determine the skin qualities consumer associate with oxidation‐related facial skin aging, and the perceived solution to oxidation‐related facial skin aging.

## 2 Methods

### 2.1 Study Design and Setting

This study was performed in female volunteers who met the inclusion/exclusion criteria. Ethics approval was not sought from an Institutional Review Board (IRB) since ethics approval is not required for consumer tests in China. All participants provided informed consent prior to taking part in the study.

### 2.2 Qualitative Focus Group Discussions

To understand how consumers perceive oxidation‐related facial skin aging effects, and their perceived solutions to it, four separate focus group discussions were held. Each focus group was comprised of six participants from Shanghai, China (*n* = 24 participants in total). The focus groups were comprised of 25–34 and 35–50 year‐old participants and were held between 13 and 26 September 2024. The focus group discussions were conducted by KANTAR consumer research agency which selected participants based on their awareness, concerns and knowledge level on five common facial skin conditions: skin puffiness, oxidation, glycation, altered skin metabolism, and reduced turnover. The respondents were asked for their understanding of these skin conditions on skin aging. Only those who were aware and able to articulate these skin effects were recruited. Participants were further identified based on their routine skincare brands and products. Main inclusion and exclusion criteria, brand and product criteria are provided in Table 1.

**TABLE 1:** Participant inclusion and exclusion criteria for (A) the focus group discussions, and (B) the online survey study.

**(A) Focus group discussions—participant inclusion criteria**	
Age	Aged 25–50 years
Gender	Female
Education level	College or higher
Residency	Local resident of Shanghai, or has lived in the city for ≥ 3 years
Brand & product user criteria	Luxury Brand users Brands including Estee Lauder, Lancôme, Sisley, SKII, Dior, Clarins, Guerlain, etc. Premium Brand users Brands & product line including Estee Lauder Re‐nutriv Ultimate lift, Lancôme ABSOLUE, HR Re‐Plasty Age Recovery, SKII luxury Pitera, LA MER, etc.
Skin type	Any skin type was included, including sensitive skin (note: a mix of different skin types for each group was included, including at least 2 sensitive skin users for each group)
Skincare usage	Current Luxury or Premium skincare brands user Uses minimum of 4 skincare product categories regularly (toner, serum, cream, emulsion, eye care, etc.) for more than 5 times per week
Makeup usage	Uses minimum of 4 makeup product categories regularly (foundation, eye shadow, contour, etc.) for more than 3 times per week
Other	Skincare product users, purchaser and main decision maker Must be aware of the following 5 skin conditions, and be concerned, and with knowledge of, at least 3 of them: oxidation, skin puffiness, glycation, altered skin metabolism, and reduced turnover Must not be pregnant or breast‐feeding, and must not have self‐reported dermatological problems
**(B) Online survey study—participant inclusion criteria**	
Gender	Female
Skin type	Any skin type was included, including sensitive skin
Skincare usage	Use luxury and premium skincare, commonly used at least 3 categories, frequency at least 5 times a week
Makeup usage	At least 3 categories, frequency at least 3 times a week
Income	Monthly household income ≥ Renminbi (RMB) 5000
Other	Decision maker of skincare and makeup

During the focus groups, the same moderator interacted with the participants by asking them to discuss their current facial skin type, and to describe factors that they considered are necessary to achieve the ideal skin condition for their age. Participants were then asked to describe perceived solutions to five common facial skin conditions: skin puffiness, oxidation, glycation, altered skin metabolism, and reduced turnover. The discussion guide used by the moderator to facilitate the qualitative focus group discussions is provided in Figure S1. This study only presents the findings on oxidation‐related skin aging.

The findings of the focus group discussions were summarized to identify the perceived effects of skincare products on oxidation‐related skin aging and the associated descriptive categories based on key messages (i.e., those that were repeated a few times by the participants), and points that participants emphasized which were related to the key messages.

### 2.3 Online Survey

Findings on the perceived effects of skincare products on oxidation‐related skin aging from the qualitative focus group were used to design an online survey that aimed to understand general consumers' skincare usage and attitude toward skincare. The survey was also designed to determine which facial skin qualities they associate with oxidation‐related skin aging, and to associate these qualities to commonly perceived skincare features. This was a self‐administered online questionnaire completed by women aged between 20 and 50 years who lived in one of four Chinese cities (Shanghai, Beijing, Chengdu, and Xi'an). A total of 800 women completed the survey, 200 from each city, with equal distribution across three age groups (20–29, 30–39, and 40–50 year‐old). The online survey was conducted on the survey platform (KANTAR‐Power CX Platform) owned by KANTAR consumer research agency which recruited participants from their database based on the inclusion criteria listed in Table 1. The online questionnaire is provided in Figure S2, which mainly includes single‐choice questions, multiple‐choice questions, and open‐ended questions.

### 2.4 Data Analyses

#### 2.4.1 Total Unduplicated Reach and Frequency (TURF) Analyses

TURF analysis was used to identify the optimal combination of attributes that maximize audience reach while minimizing overlap. The process involved creating a binary matrix where each respondent's preference or selection was recorded. Iterative simulations were then performed to evaluate various attribute combinations and their reach. The optimal solution path was then identified, where (i) the starting attribute covered the highest percentage of customers; (ii) the next attribute was selected to maximize cumulative reach; and (iii) the process continued iteratively to form an optimal combination path. TURF analyses were performed using Quick TURF software.

#### 2.4.2 Principal Component Analyses (PCA)

PCA analyses were performed to visualize relationships between attributes and visualize patterns in the data. PCA analyses were performed using ToolBox, an Excel add‐on designed for PCA.

### 2.5 Statistical Analyses

For the online survey, data were analyzed by summarizing different comparison groups, including demographics (age, city tiers, individual income, user type (luxury vs. premium)) and skincare needs (anti‐oxidation, repairing, nourishing, etc.).

To assess statistical significance, Chi‐squared tests (using SPSS software) were used to compare differences between groups and determine whether the observed variations were statistically meaningful under 95% confidence level.

## 3 Results

### 3.1 Qualitative Focus Group Discussions

A summary of the demographics of the women who took part in the qualitative focus group discussions (*n* = 24 participants) is provided in Figure S3. All 24 participants identified oxidation as one of their skin concerns.

#### 3.1.1 Perceived Causes and Effects of Oxidation‐Related Skin Aging

**FIGURE 1:**
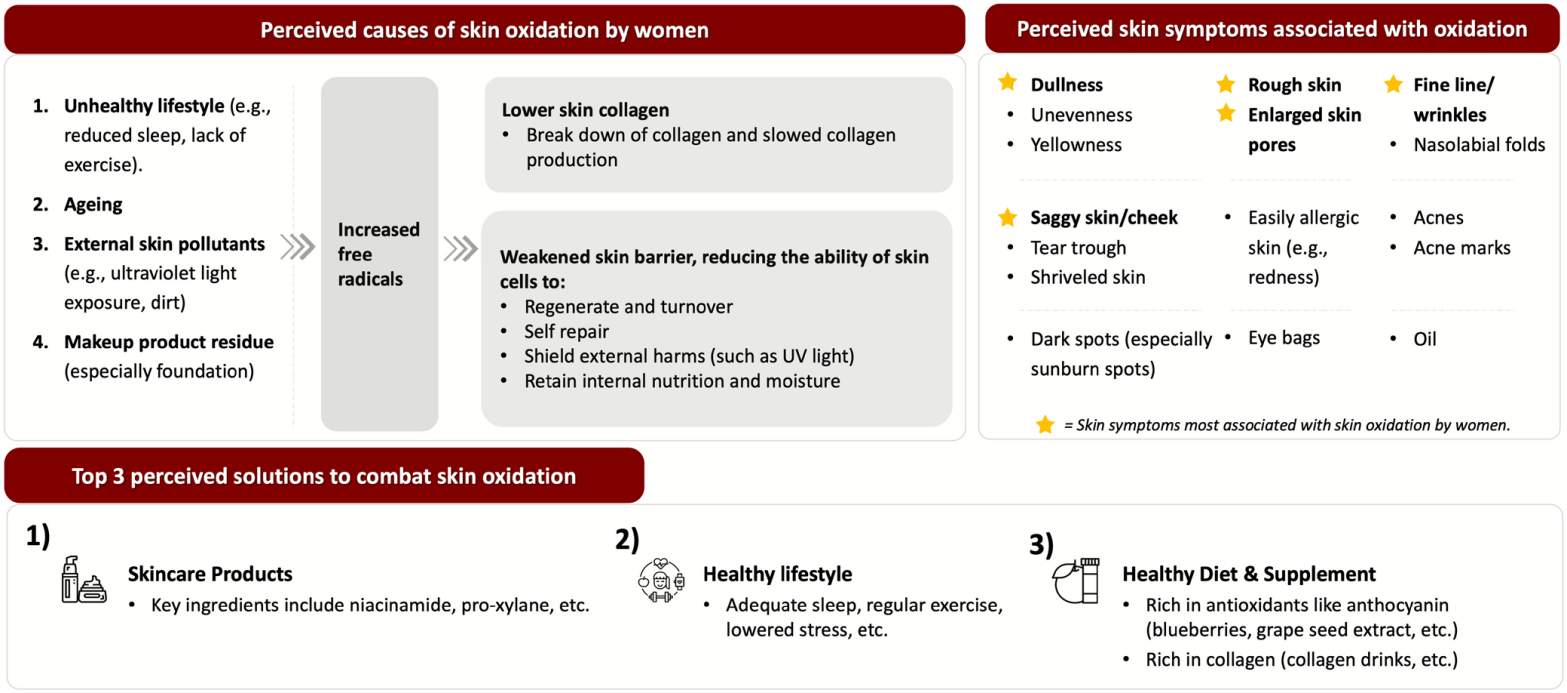
Focus group discussions revealed perceived causes and effects of oxidation, skin symptoms associated with oxidation, and perceived solutions to combat skin oxidation.

Qualitative focus group discussions found that women perceived oxidation‐related skin aging effects to be caused by unhealthy lifestyle, aging, external pollutants, and makeup residue, which leads to a weakened skin barrier and impaired collagen stimulation (Figure 1).

#### 3.1.2 Perceived Symptoms of Oxidation‐Related Skin Aging

Participants associated several skin features to be caused by oxidation (Figure 1). Among the list of perceived skin symptoms mentioned in the focus group discussions, the participants identified that the five skin symptoms most associated with skin oxidation were dullness, rough skin, enlarged skin pores, fine lines/wrinkles, and sagging skin/cheeks (Figure 1).

#### 3.1.3 Perceived Solutions to Combating Oxidation‐Related Skin Aging

Participants considered skincare products to be the primary solution to managing skin oxidation effects (Figure 1), however they also cited a healthy lifestyle (involving adequate sleep and regular exercise), a healthy diet rich in antioxidants, and supplements to be factors that can combat skin oxidation.

#### 3.1.4 Perceived Effects of Skincare on Oxidation‐Related Skin Aging

Participants were asked how skincare products would help to reduce the visible signs of oxidation. They were also asked to associate the perceived effects to specific descriptive categories that are commonly related with skincare product claims. Participants most strongly perceived skincare to improve brightness, radiance, and refine skin texture; these perceived results are associated with the descriptive category “skin and spot lightening,” which was the top ranked category (Table 2). The second highest category identified was “firming.” Other descriptive categories that participants identified can be ameliorated with skincare were “sun protection,” “nourishing,” “repairing,” and “hydration” (Table 2).

**TABLE 2:** The perceived effects of anti‐oxidation skincare by focus group participants, and the descriptive categories associated with perceived effects.

Perceived effects of skincare	Descriptive category associated with perceived effects
Brightness Radiance Fine texture Even skin tone Fair and clear skin	Skin and spot lightening^a^
Firmness	Firming^a^
Fine texture Even skin tone	Sun protection
Plumping Tightening pores	Nourishing
Acne marks corrected Healthy skin (not easily sensitive) Reduced redness	Repairing
Hydrated Moisture‐sebum balanced	Hydration

^a^ Top associated descriptive categories associated with skin oxidation.

### 3.2 Large‐Scale Online Survey

**FIGURE 2:**
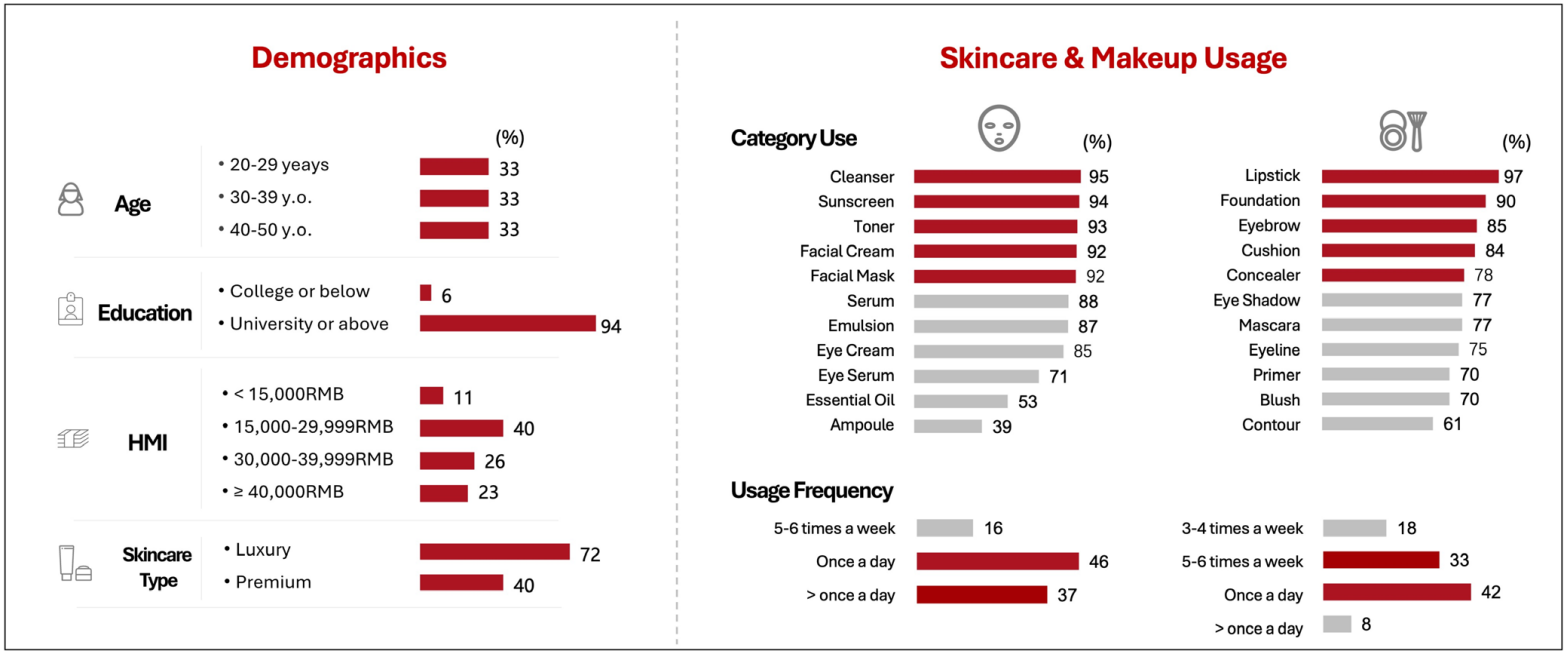
A summary of the demographics and skincare and makeup usage of the 800 online survey respondents. Numbers next to bars denote the percentage (%) of participants; A total of 800 respondents completed the survey and were included in this analysis; MHI, monthly household income; RMB, Renminbi.

The average time to complete the survey was 25 min. A summary of the demographics and skincare/makeup usage of the survey participants is summarized in Figure 2.

#### 3.2.1 Skincare Needs

**FIGURE 3:**
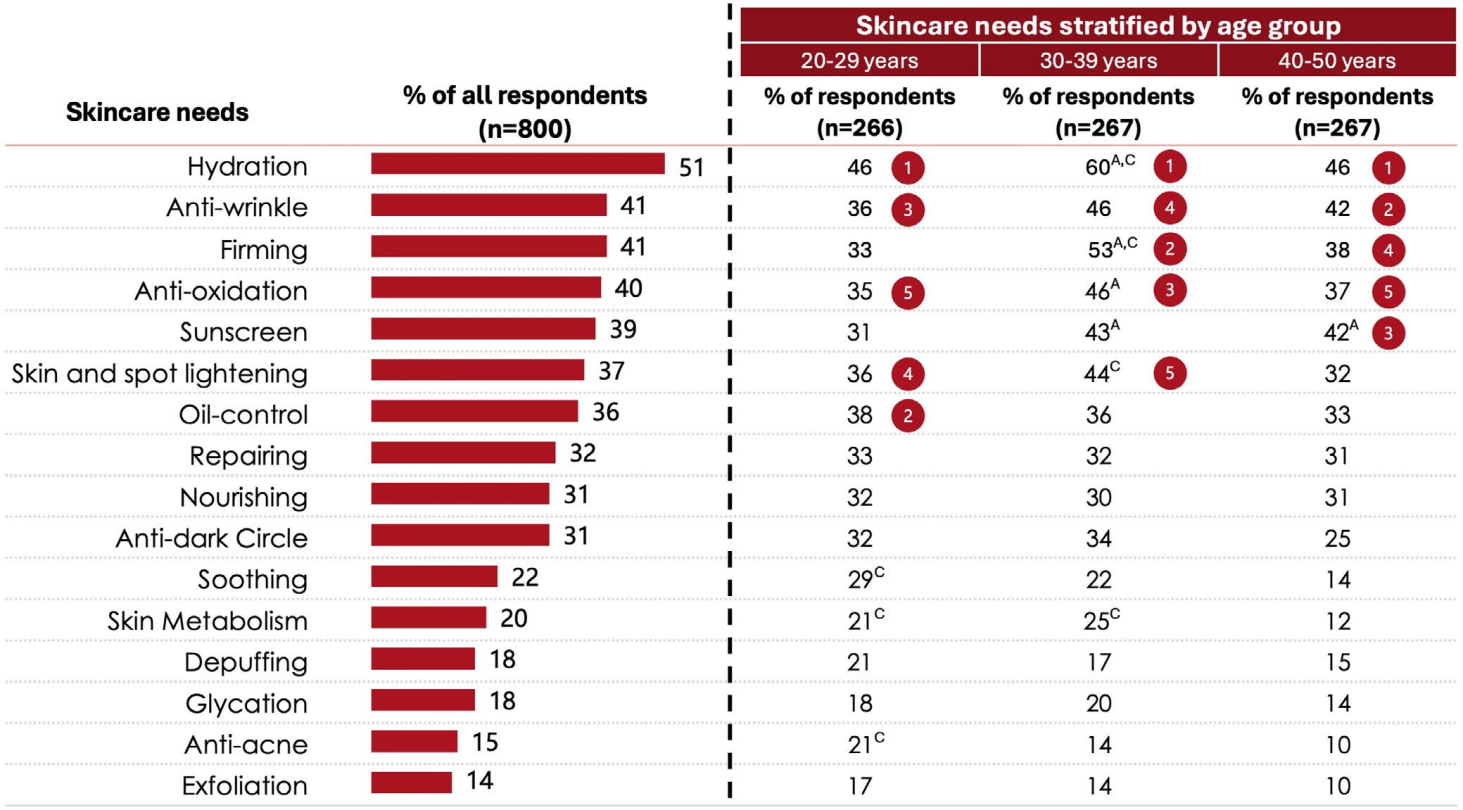
Skincare needs of survey respondents. ^A^Significantly higher than the 20–29 years age group as determined by Chi‐squared analysis (*p* ≤ 0.05). ^C^Significantly higher than the 40–50 years age group as determined by Chi‐squared analysis (*p* ≤ 0.05). The numbers shown within a red circle denote the top five skincare needs within that age group.

The skincare needs were surveyed. Respondent skincare needs are summarized in Figure 3. Skincare needs were found to be focused on hydration and antiaging. Hydration was the most selected skincare need across all age brackets. Of the 800 respondents, 51% selected hydration as a skincare need. This was followed by anti‐wrinkle (41%); firming (41%); anti‐oxidation (40%); sunscreen (39%); skin and spot lightening (37%); and oil control (36%).

Respondents in the 30–39 years age bracket tended to have relatively more skincare needs than respondents in the other two age brackets; significantly greater proportions of 30–39 year‐old respondents indicated skincare needs for hydration, firming, anti‐oxidation, and sunscreen than the 20–29 year‐old respondents (*p* ≤ 0.05) (Figure 3). Additionally, more 30–39 year‐old respondents reported a need for hydration, firming, skin and spot lightening, and skin metabolism than the 40–50 year age group (*p* ≤ 0.05) (Figure 3). More respondents in the 20–29 year age bracket indicated a need for soothing, skin metabolism, and anti‐acne skincare than respondents in the 40–50 year age brackets (*p* ≤ 0.05) (Figure 3). More respondents in the 40–50 year age bracket indicated a need for sunscreen than those in the 20–29 year age bracket (*p* ≤ 0.05) (Figure 3).

#### 3.2.2 Perceived Association Between Skincare Product Features and Reduced Oxidation‐Related Skin Aging

**FIGURE 4:**
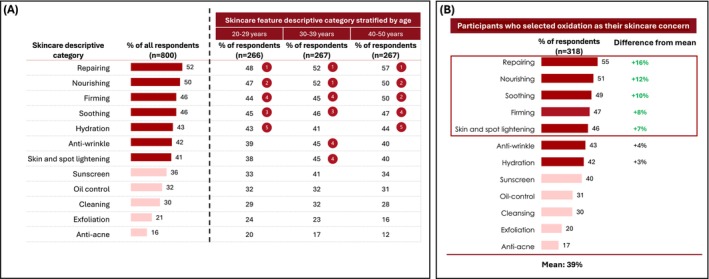
Skincare feature descriptive categories that were associated with reduced oxidation by online survey respondents. The numbers shown within a red circle denote the top five skincare needs within that age group. Chi‐squared analysis found no statistically significant differences between the three age groups (*p* > 0.05).

Survey respondents were asked to select skincare efficacies that they believed were associated with combating oxidation (summarized in Figure 4A). The perceived skincare efficacies most associated with reducing oxidation were repairing (selected by 52% of respondents); nourishing (50%); firming (46%); soothing (46%); hydration (43%); anti‐wrinkle (42%); and skin and spot lightening (41%). The differences between the three age groups were not statistically significant (*p* > 0.05).

Sub‐group analysis of the 318 participants who selected oxidation as one of their skincare concerns found that, for these respondents, soothing and skin & spot lightening ranked higher while firming and hydration ranked lower than the total survey population of 800 participants (Figure 4B).

#### 3.2.3 Skincare Product Usage and Satisfaction

**FIGURE 5:**
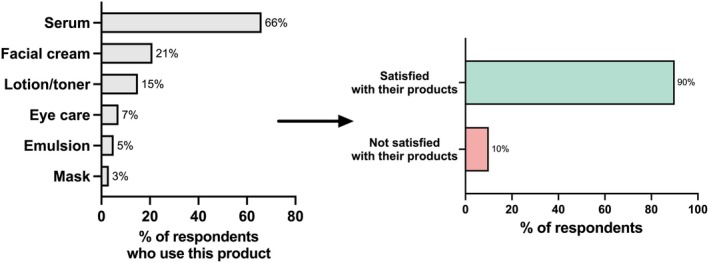
Skincare products that survey respondents with skin oxidation concerns (*n* = 309) use to combat skin oxidation.

Of the respondents who had skin oxidation concerns (*n* = 318), approximately 97% (*n* = 309) used skincare as a solution to oxidation. Two‐thirds (66%) of the respondents who had skin oxidation concerns used serums for antioxidant purposes; 21% used facial creams; 15% used lotion/toner; 7% used eye care; 5% used an emulsion; and 3% used masks for antioxidant purposes (Figure 5). The satisfaction rate with the products used was 90%. The main reasons for dissatisfaction in the remaining 10% of participants were a perceived lack of effectiveness and concerns about the safety of the ingredients.

#### 3.2.4 Skincare Product Descriptive Categories and Consumer Reach

**FIGURE 6:**
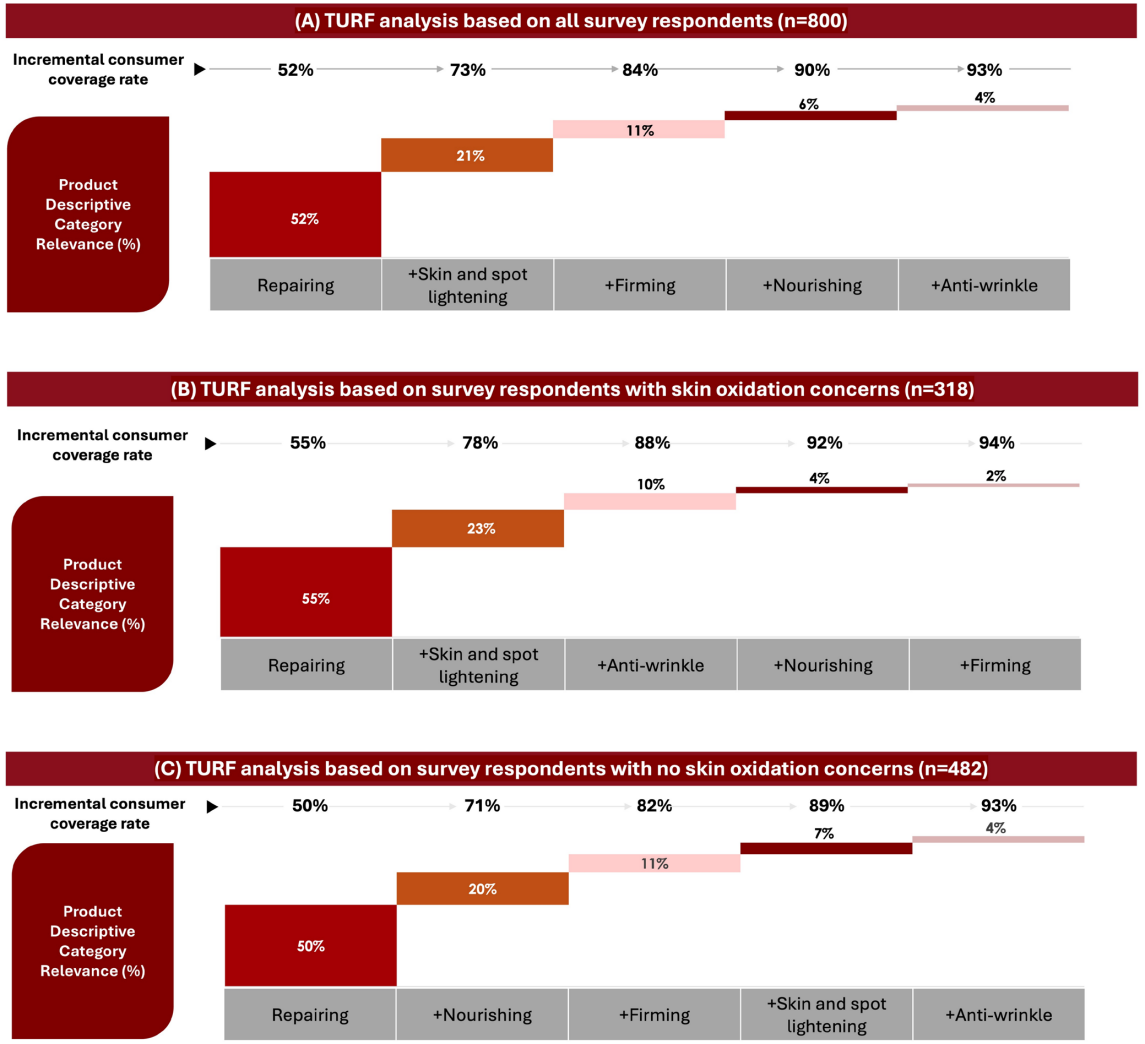
TURF analysis shows the combination of five skincare product descriptive categories that have the highest consumer reach for oxidation. (A) shows TURF analysis results based on data from all survey respondents (*n* = 800), (B) shows results based on respondents who indicated skin oxidation concerns (*n* = 318), and (C) shows results based on respondents who did not indicate skin oxidation concerns (*n* = 482). TURF, total unduplicated reach and frequency.

Based on the biochemical mechanisms reported in literature, oxidative stress on the skin can lead to impaired skin barrier, skin tone unevenness, pigmentary disorder, skin roughness and wrinkles [1, 3]. These skin physiological aspects were then associated with the five descriptive categories on “skin repairing,” “skin and spot lightening,” “firming,” “nourishing,” and “antiwrinkle” based on the expected skin effects of each category. These associations were made by us based on the expected skincare effects of each category. For instance, a product with “skin repairing” effect was linked to skin barrier improvement, while a product with “skin and spot lightening” effect was associated with improving skin tone evenness and pigmentary disorder. The TURF analysis was used to understand the relevance of these five descriptive categories to oxidation. A combination of these five descriptive categories was shown to have a consumer reach of 93% (Figure 6A), among which, repairing, skin and spot lightening, and firming, were most differentiated categories to boost coverage of the surveyed consumer population. The product descriptive category with the greatest individual consumer reach when selected as the starting point was “repairing;” this category was the most selected by consumers (52%) as the parameter perceived as most related to oxidation. The TURF analysis indicated that a further 21% of female consumers can be reached with the category “skin and spot lightening” (Figure 6A).

Subgroup TURF analyses were performed on data from the 318 respondents who expressed skin concerns relating to skin oxidation (Figure 6B) and on data from the 482 respondents who did not express concerns relating to skin oxidation (Figure 6C). The ranking of skin and spot lightening was lower among the subgroup of people who do not have oxidation concerns (Figure 6C) than those who have oxidation concerns (Figure 6B), indicating that this group does not associate anti‐oxidation with skin and spot lightening as closely.

#### 3.2.5 PCA Analysis

**FIGURE 7:**
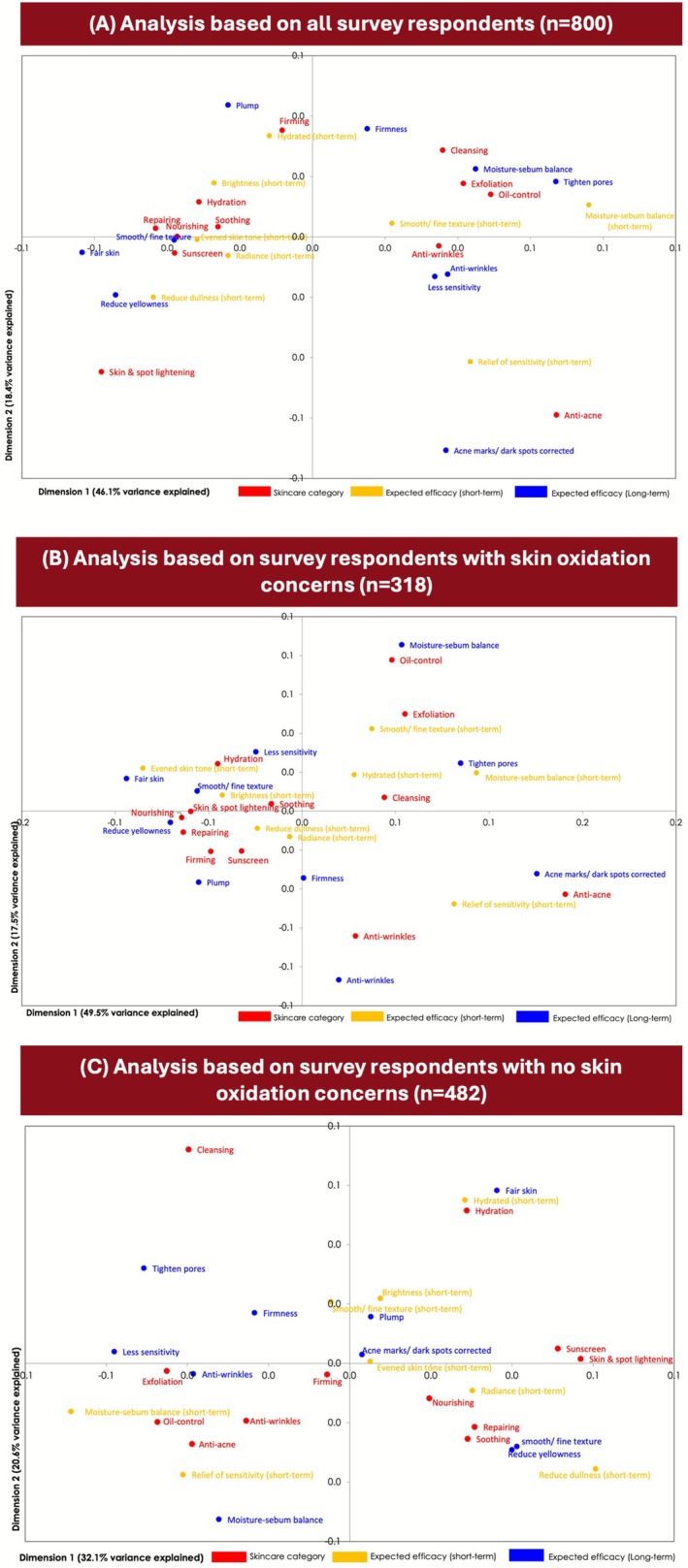
Principal component analysis (PCA) of the online survey data show the level of association of descriptive categories (red) with short‐term (yellow) and long‐term (blue) expected skincare efficacies based on data from all survey respondents (A), respondents who expressed oxidation concerns (B), and respondents who did not express oxidation concerns (C). Red dots represent key skincare descriptive categories. The closer the distance between skincare categories, the more similar the expected efficacy they share. Blue dots represent long‐term expected efficacy. The closer a blue dot is to a descriptive category (red dot), the more relevant the blue dot is to that category.

Of the 12 skincare descriptive categories analyzed (shown in red; Figure 7A), three descriptive categories of “skin and spot lightening,” “anti‐acne,” and “firming” were found to be mutually exclusive. Respondents were asked to select their short (shown in yellow) and long term (shown in blue) expected skincare efficacies relating to reducing oxidation. As shown in Figure 7A, the expected efficacies most related to the descriptive category “skin and spot lightening” were reducing skin yellowness and dullness, followed by radiance and fair skin. The descriptive category “sunscreen” shared similar efficacies to “skin and spot lightening,” indicating that they were perceived similarly. The descriptive categories “cleansing,” “exfoliation,” and “oil control” were less differentiated, as they shared similar expected efficacies to achieve moisture‐sebum balance, tightened pores, and smooth texture. The descriptive categories “repairing,” “nourishing,” “soothing,” and “hydration” were also clustered, with no clearly differentiated expected efficacies associated (Figure 7A).

Subgroup PCA analyses were performed on data from the 318 respondents who expressed skin concerns relating to skin oxidation (Figure 7B) and on data from the 482 respondents who did not express concerns relating to skin oxidation (Figure 7C). PCA analysis for those with oxidation skin concern (Figure 7B) exhibited close clustering of skin & spot lightening, nourishing, and repairing; these skincare categories shared the same efficacy on reducing yellowness. Conversely, clustering of these skincare categories was not as close for those without oxidation skin concerns (Figure 7C).

## 4 Discussion

The objective of this study was to gain a better understanding of female consumer perceptions in four Chinese cities toward effects of oxidation‐related facial skin aging, and the efficacies of skincare products they perceived to combat oxidation‐related skin aging.

Qualitative focus group discussions with small groups of female consumers with skin oxidation concerns found that these women predominantly associated five skin symptoms (dullness, rough skin, enlarged pores, fine lines/wrinkles, and sagging skin/cheeks) with oxidation. This consumer group perceived several skincare product efficacies to act via anti‐oxidation mechanisms. These efficacies include improving skin firmness, brightness, radiance, fine texture, tone, clarity, and fairness. These effects were associated by women primarily with the descriptive skincare categories “skin and spot lightening” and “firmness,” indicating that women with skin oxidation concerns believe skincare with anti‐oxidation benefits predominantly lightens and tightens the skin.

Online survey results found that consumers generally associated anti‐oxidation with the skincare descriptive categories of “repairing,” “nourishing,” “firming,” and “soothing.” “Repairing” was the skincare category most associated with anti‐oxidation across all age groups, followed by “nourishing.” Other strongly associated descriptive categories across all age groups were “firming,” “soothing,” “hydration,” “anti‐wrinkle,” and “skin and spot lightening.” It was not surprising that these skincare product features were associated with reducing oxidation since oxidation has been shown to exacerbate skin tone unevenness, pigmentary disorder, skin roughness and wrinkles [3]. Oxidative damage to proteins, lipids, and DNA in skin cells can impact collagen, elastin, and matrix metalloproteases [1], impacting firmness and promoting wrinkle formation. Impaired cellular autophagy and increased senescence lead to skin barrier disruption, which impacts skin hydration and repair [4]. Furthermore, oxidation can promote melanogenesis, potentially leading to hyperpigmentation and impacting skin tone [5].

TURF analysis is often used by enterprises to optimize the strategies of their products in order to maximize consumer reach. In this study, TURF analysis of the survey findings identified an optimal combination of five skincare product descriptive categories (namely “repair,” “skin and spot lightening,” “firming,” “nourishing,” and “anti‐wrinkles”) that yield the greatest unduplicated consumer reach of 93%. This descriptive category combination is expected to appeal to the largest possible consumer population while minimizing overlap. The categories “repair,” “skin and skin and spot lightening,” and “firming” were the most differentiated categories to boost the coverage of population since a combination of these three categories yielded an expected population reach of 84%. Interestingly, this study's findings showed that 52% of surveyed female consumers can be reached using only the “repair” descriptive category, indicating that consumers primarily associate antioxidant products with skin repair. This was further illustrated using PCA analysis, which showed clustering of “repair” with another key descriptive category (nourishing) found to be highly associated with anti‐oxidation.

A key finding from this study was that study participants with oxidation concerns had a stronger association between anti‐oxidation and “skin and spot lightening” than consumers without oxidation concerns. Qualitative focus group discussions with small groups of female consumers with skin oxidation concerns found that these women primarily associated anti‐oxidation with the descriptive skincare categories “skin and spot lightening” and “firmness;” this suggested that women with skin oxidation concerns believed skincare with anti‐oxidation benefits predominantly lightens and tightens the skin. Subgroup analysis from the larger online survey supported this since “skin and spot lightening” was more strongly associated with anti‐oxidation by consumers who had skin oxidation concerns than those who did not have skin oxidation concerns. These findings were further supported by the TURF and PCA subgroup analyses, which found a higher ranking for “skin and spot lightening” among women with skin oxidation concerns. Overall, these findings suggested that women with concerns about skin oxidation had a better understanding of the association between anti‐oxidation and skin and spot lightening, and these consumers viewed this skincare attribute with higher priority.

Another key insight into consumer behavior gained from this study was that almost all (97%) of consumers surveyed used skincare to combat skin oxidation. This demonstrates that consumers have a strong perception that antioxidant products are efficacious, a finding further supported by a 90% satisfaction rate with antioxidant products. The most commonly used type of antioxidant skincare used is serum, with two‐thirds of women using a serum for antioxidant purposes.

Limitations to this study include the lack of sufficient data in the large‐scale study to validate other key findings identified in the focus group discussions. For instance, participants associated enlarged skin pores as one of the perceived symptoms of oxidation‐related skin aging, however this was not clearly demonstrated in the PCA analysis. Future research includes expanding the surveys to other demographics to fully validate the consumer perceptions identified in this study.

In conclusion, key findings from this study were that consumers mainly associate anti‐oxidation with skincare with that has the descriptive categories “repair” and “skin and spot lightening.” These associations were stronger in consumers who had skin oxidation concerns than those who did not. This study found 73% of the online survey participants would be reached by skincare products with the descriptive categories “repair” and “skin and spot lightening,” hence suggesting that these skincare product features have the potential to reach a sizeable proportion of skincare consumers. These findings can be used to develop consumer‐oriented facial skin care products, and to improve the quality features of products designed to combat skin oxidative aging effects.

## Author Contributions

**Bee Leng Lua:** test conception and design. **Sharon Shi:** data collection, analysis. **Kelly Chua:** scientific substantiation, efficacy claims.

## Funding

This study was funded by Groupe Clarins, Singapore. Medical writing support was funded by Groupe Clarins, Singapore, in accordance with Good Publication Practice (GPP3) guidelines (http://www.ismpp.org/gpp3).

## Ethics Statement

Ethics approval was not sought from an Institutional Review Board (IRB) since ethics approval is not required for consumer tests in China. All participants provided informed consent prior to taking part in the study.

## Conflicts of Interest

K.C., B.L.L., and S.S. were employees of Groupe Clarins, Singapore, at the time of the study.

## Supporting information


**Figure S1:** Guide used by moderators for qualitative focus group discussions.
**Figure S2:** Online questionnaire.
**Figure S3:** A summary of the demographics of the women who took part in the qualitative focus group discussions (*n* = 24 participants).

## Data Availability

The data that support the findings of this study are available on request from the corresponding author. The data are not publicly available due to privacy or ethical restrictions.
