# Supplementary material for: Consumer Perceptions of Oxidation‐Related Effects on Facial Skin Aging and Skincare Product Features
Source: J Cosmet Dermatol. 2025 Dec 19;24(12):e70623. doi: 10.1111/jocd.70623 (PMC12717496; doi:10.1111/jocd.70623)

**SUPPLEMENTARY MATERIAL**

**Supplementary Figure 1: Guide used by moderators for qualitative focus group discussions**

**Skincare Secondary Claims Study**

| *Note: This document serves as a guide to the moderator. The discussion may not follow in the same flow; or questions may be asked in a different way during the actual discussion. The moderator may paraphrase and/or change the flow of the discussion to suit the interview dynamics and/or the context/ culture where the group is held in if it better addresses the research objectives.* |
| --- |

**DISCUSSION OUTLINE**

| **Section** | **Time (120mins)** | **Objectives** |
| --- | --- | --- |
| 1. Introduction & warm-up | 5 mins | - Introduce the objectives of the discussion and house-keeping rules - Build good rapport with the respondents |
| 1. Current skin concerns and ideal skin condition | 10 mins | - Understand their skin conditions and concerns - Identify their ideal skin condition |
| 1. Perception and attitude toward the key claims | 95 mins | **For Depuffing/Oxidation/Glycation/Skin Metabolism/ Exfoliation**   - Understand the causes/ working mechanism of each claim direction - Understand their solutions (esp. on skincare) towards different issues - For each claim direction, understand their product association (skincare & makeup), usage and efficacy satisfaction |
| 1. Tag-on (Perception toward other claims) | 10 mins | - Understand their perceptions toward tag-on claims |
| 1. Wrap Up | - | - Check additional questions - Thanks respondents and end the discussion |

**DETAILED QUESTIONS FOR DISCUSSION**

**PART 1. Warm up & introduction [5 mins]**

**Moderator to introduce,**

- - Encourage respondent to be open and honest, no right or wrong answers and free exchange of ideas and expressions.
  - Confidentiality, duration about 2 hours

**Respondent’s introduction:**

- - About self: name, age, occupation, hobbies…

**PART 2. Current skincare concerns and ideal skin condition [10 mins]**

**Today’s topic is about skincare, first I’d like to know how you feel about your skin.**

**Current skin concerns**

- - How would you perceive your skin type? How can you tell? *–e.g., dry, oil, combination, etc.*
  - What skin concerns do you have?
    - *e.g., dullness, wrinkles, dark sports, dryness, etc.*
    - [For sensitive skin] what specific symptoms do you experience? In what occasion and how frequently do these symptoms usually occurs?

**Ideal skin condition**

- - What is the ideal condition (at your age) that you want to achieve? *– e.g., radiance, firm skin, etc.*
    - Among all, what do you perceive are the hardest to achieve? Why?

**PART 3. Perception and attitude toward the key claims [95 mins]**

Moderator probe for perception and expectation toward the 5 key claims: Depuffing去水肿，Oxidation氧化，Glycation糖化，Skin Metabolism肌肤新陈代谢，Exfoliation去角质

| **Depuffing/去水肿** |
| --- |

**Symptoms**

- - What is your understanding of skin puffing?
  - Do you have puffing issue? How could you identify?
    - **[Associated skin condition]** Could you describe the skin condition (and specific area) when have relevant issue? How does it look like? (focus on top symptoms)
      - - *e.g., skin thickness in upper eyelids…*
    - How severe do you think it is (light/mid/severe)? How do you define?

**Solution & Expectations**

- - What are the solutions for puffing issue?
    - Skincare products vs other solutions (makeup products, massage with beauty devices, lifestyle adjustment such as coffee/diet therapy/sleep, physical exercise, etc.)
    - What is the role and expectation toward skincare products as solution?
      - - How skincare helps to achieve depuffing?

Moderator to focus on skincare products and probe each one:

- - What skincare products do you use to deal with puffing issues?
    - *-e.g., Clarins V shaping lift serum*
  - What problems does this product help to solve?
    - To link back with associated skin conditions mentioned above
  - Why do you think this product helps with these issues?
  - **[Expected results]** What kind of benefits/exact changes do you expect to happen on your face?

| **Oxidation/氧化** |
| --- |

**Symptoms**

- - What is your understanding of skin oxidation?
  - Do you have skin oxidation issue? How could you identify (visually)?
    - **[Associated skin condition]** Could you describe the skin condition when have relevant issue? How does it look like?
      - - *e.g., skin dullness, dryness, low elasticity…*
    - In what occasion and how frequently do these issues usually happen?
      - - *e.g., after using makeup products (esp. foundation) …*

**Solution & Expectations**

- - What are the solutions for oxidation issue?
    - Skincare products vs other solutions (e.g. nutrition supplements, etc.)
    - What is the role and expectation toward skincare products as solution?
      - - How skincare helps to solve oxidation issue?

Moderator to focus on skincare products and probe each one:

- - What skincare products do you use to deal with oxidation issues?
  - What problems does this product help to solve?
    - To link back with associated skin conditions mentioned above
  - Why do you think this product helps with these issues?
  - **[Expected results]** What kind of benefits/exact changes do you expect to happen on your face?

| **Glycation/糖化** |
| --- |

**Symptoms**

- - What is your understanding of skin glycation?
  - Do you have skin oxidation issue? How could you identify?
    - **[Associated skin condition]** Could you describe the skin condition when have relevant issue? How does it look like?
      - - *e.g., dark spots, yellowness, sagging skin, skin is not firm…*

**Solution & Expectations**

- - What are the solutions for glycation issue?
    - Skincare products vs other solutions (dietary therapy, etc.)
    - What is the role and expectation toward skincare products as solution?
      - - How skincare helps to solve oxidation issue?

Moderator to focus on skincare products and probe each one:

- - What skincare products do you use to deal with glycation issues?
  - What problems does this product help to solve?
    - To link back with associated skin conditions mentioned above
  - Why do you think this product helps with these issues?
  - **[Expected results]** What kind of benefits/exact changes do you expect to happen on your face?

| **Skin Metabolism/肌肤新陈代谢** |
| --- |

**Symptoms**

- - What is your understanding of skin metabolism?
  - Do you have the needs? How could you identify?
    - **[Associated skin condition]** Could you describe the skin condition (and specific area) when perceived need the skin metabolism? How does it look like?
      - - *e.g., dullness, rough texture…*

**Solution & Expectations**

- - What are the solutions for boosting skin metabolism?
    - Skincare products vs other solutions
    - What is the role and expectation toward skincare products as solution?
      - - How skincare helps to solve metabolism issues?

Moderator to focus on skincare products and probe each one:

- - What skincare products do you use to deal with metabolism issues?
  - What problems does this product help to solve?
    - To link back with associated skin conditions mentioned above
  - Why do you think this product helps with these issues?
  - **[Expected results]** What kind of benefits/exact changes do you expect to happen on your face?

| **Exfoliation/去角质** |
| --- |

**Symptoms**

- - What is your understanding of skin exfoliation?
  - Do you have the needs? How could you identify?
    - **[Associated skin condition]** Could you describe the skin condition (and specific area) when perceived need the skin exfoliation? How does it look like?
      - - *e.g., dullness, rough texture, skincare products being hard to absorbed…*

**Expectations**

- - After using skincare products that helps exfoliation,
    - **[Expected result]** What kind of benefits/ exact changes do you expect to happen on your face?
    - Moderator to probe if not mentioned below
      - - reduce roughness/smoother skin使肌肤光滑，不粗糙
        - improve/refine skin texture, resurface the skin改善肌肤质地
        - radiance/brightness 肌肤有光泽，提亮
        - skin is renewed/regenerated 焕肤，肌肤焕新

**PART 4. Tag-on (Perception toward other claims) [10 mins]**

Below are the tag-on claims to check at the end. Moderator may skip the claims that are already covered in previous discussion.

**Anti-dark circle 祛黑眼圈**

- - Do you have dark circle problems? What are the relevant efficacies to deal with it and why? (probe if not mentioned **whitening**)
  - What skincare products you use to deal with anti-dark circle problems?

**Comfort** **使肌肤舒适**

- - How would you perceive it? How could you identify?
    - **Sensorial vs emotional**
    - Moderator to see if any difference between sensitive skin users vs other users
  - What are the relevant skincare efficacies to help achieve it and why? (probe if not mentioned **hydration/soothing**)

**Vitality, Energetic/energized, Stronger 肌肤有活力**

- - How would you perceive it? How could you identify?
    - **Sensorial vs emotional**
    - Moderator to check the perception on different structures of the claims “I feel energetic” and “my skin feels energetic”.
  - What are the relevant skincare efficacies to help achieve it and why?

**Lock in skin moisture 锁水**

- - What are the relevant skincare efficacies and why? (probe if not mentioned **hydration/plumpness**)
    - How do you identify for the moisture lock? *– e.g., on epidermis, on dermis, etc.*

**Prepare skin for the next step of skincare 为后续护肤步骤准备**

- - What skincare categories (and relative efficacies) are associated with it? Why?
    - Probe if not mentioned **hydration/cleansing**
    - For category to see if mentioned facial cleanser/facial sheet mask *– e.g., to use TE (精华水) on sheet mask, etc.*

**Dehydration line 干纹**

- - What are the relevant efficacies to solve it and why?

**Yellowness vs dullness 黄气vs暗沉**

- - How do you perceive yellowness/dullness? Are they the same or different?
    - For yellowness to see what related skin parameter (sebum, pores, etc.) constitute toward yellowness

**Check if clients have additional questions - End & thank the respondents.**

**Supplementary Figure 2: Online Questionnaire**

| ­**Skincare Claims Study**  **Main Questionnaire** |
| --- |

**Skin Conditions**

**K1. Skin type**

| K1 | What is your facial skin type? [Single choice] | Code | Skip |
| --- | --- | --- | --- |
|  | Dry skin | 1 |  |
|  | Combination-dry skin | 2 |  |
|  | Normal skin | 3 |  |
|  | Combination-oily skin | 4 |  |
|  | Oily skin | 5 |  |

**K01. Sensitive-skin status**

| K01 | Do you consider yourself to have sensitive skin? [Single choice] | Code | Skip |
| --- | --- | --- | --- |
|  | Yes | 1 | **K02** |
|  | No | 2 |  |

**K02. Sensitive-skin product usage**

| K02 | **Ask only if K01 = Yes**  When your skin feels sensitive, do you normally use skincare products that are specifically designed for sensitive skin? [Single choice] | Code | Skip |
| --- | --- | --- | --- |
|  | Yes | 1 |  |
|  | No | 2 |  |

**K15. Skincare needs**

| K15 | Which of the following skincare needs do you have? [Multiple choice] | Code | Skip |
| --- | --- | --- | --- |
| (R1) | Skin and spot lightening | 1 |  |
| (R2) | Suncreen | 2 |  |
| (R3) | Anti-acne | 3 |  |
| (R4) | Nourishing | 4 |  |
| (R5) | Repairing | 5 |  |
| (R6) | Anti-wrinkle | 6 |  |
| (R7) | Firming | 7 |  |
| (R8) | Soothing | 8 |  |
| (R9) | Oil-control | 9 |  |
| (R10) | Exfoliation | 10 |  |
| (R11) | Hydration | 11 |  |
| (R12) | Depuffing | 12 | **K16** |
| (R13) | Anti-oxidation | 13 | **K16** |
| (R14) | Anti-glycation | 14 | **K16** |
| (R15) | Skin metabolism | 15 | **K16** |
| (R16) | Anti-dark circle | 16 | **K16** |
|  | Others, please specify: __________ | 98 |  |

**K16. Solutions for selected skincare needs**

| K16 | **Ask only if any of K15 codes 12–16 is selected**  How do you usually address your need for [insert selected items in K15]? [Multiple choice] | Code | Skip |
| --- | --- | --- | --- |
|  | Use skincare products | 1 |  |
|  | Control diet | 2 |  |
|  | Exercise | 3 |  |
|  | Adjust sleep schedule | 4 |  |
|  | Facial massage | 5 |  |
|  | Others, please specify: __________ | 98 |  |

**H2. Hidden Question - Skincare Demand Quota**

| H2 | Skincare demand quota | | Code | Skip |
| --- | --- | --- | --- | --- |
|  | Depuffing | **K15=12 and K16=1** | 1 |  |
|  | Anti-oxidation | **K15=13 and K16=1** | 2 |  |
|  | Anti-glycation | **K15=14 and K16=1** | 3 |  |
|  | Skin metabolism | **K15=15 and K16=1** | 4 |  |

**Main Questionnaire**

**Depuffing**

**DP1. Depuffing: expected effects**

| DP1 | Which of the following effects do you believe can be achieved through depuffing? [Multiple choice] | Code | Skip |
| --- | --- | --- | --- |
| (R1) | Firming & lifting | 1 |  |
| (R2) | V-shape | 2 |  |
| (R3) | Defined jawline | 3 |  |
| (R4) | Defined contour | 4 |  |
| (R5) | Smooth with no lines | 5 |  |
| (R6) | Comfort | 6 |  |
| (R7) | Calming and soothing | 7 |  |
| (R8) | Plump | 8 |  |
| (R9) | High skin turgor | 9 |  |
| (R10) | Elastic skin | 10 |  |
| (R11) | Reduce eye puffiness | 11 |  |
| (R12) | Reduce swelling | 12 |  |
| (R13) | Reduce eye fatigue | 13 |  |
|  | Others, please specify: __________ | 98 |  |

**DP2. Depuffing: category relevance**

| DP2 | Which skincare benefit categories do you associate with depuffing? [Multiple choice] | Code | Skip |
| --- | --- | --- | --- |
|  | Nourishing | 4 |  |
|  | Repairing | 5 |  |
|  | Anti-wrinkle | 6 |  |
|  | Firming | 7 |  |
|  | Soothing | 8 |  |
|  | Others, please specify: __________ | 98 |  |
|  | None of the above | 99 |  |

**DP3. Depuffing: expected time**

| DP3 | How soon do you expect to see depuffing effects after using a skincare product? [Single choice] | Code | Skip |
| --- | --- | --- | --- |
|  | Immediately | 1 |  |
|  | Within half a day | 2 |  |
|  | Within 1 day | 3 |  |
|  | Within 1 week | 5 |  |
|  | More than 1 week | 6 |  |

**DP4-DP6** **ask only if H2=1**

**DP4. Depuffing: product usage**

| DP4 | Which skincare products do you use to address puffiness? Please write at least one brand and product name.  Brand 1: __________ Product name 1: __________  Brand 2: __________ Product name 2: __________  Brand 3: __________ Product name 3: __________ |
| --- | --- |
|  |  |

**DP5. Depuffing: satisfaction**

| DP5 | Overall, how satisfied are you with the skincare products you currently use for puffiness? Rate 1-5, 5=very satisfied,1=very unsatisfied. [Single choice] | Code | Skip |
| --- | --- | --- | --- |
|  | 5 | 5 |  |
|  | 4 | 4 |  |
|  | 3 | 3 |  |
|  | 2 | 2 |  |
|  | 1 | 1 |  |

**DP6. Depuffing: low satisfaction reason**

| DP6 | **Only if DP5=1-3**  What are the reasons for your low satisfaction with the products you use for puffiness? [Open-ended]  _________________________________________________________________________________ |
| --- | --- |

**Oxidation**

**O1. Oxidation: expected effects**

| O11 | Which of the following effects do you believe can be achieved through anti-oxidation within a short term (within one week)? [Multiple choice] | Code | Skip |
| --- | --- | --- | --- |
| (R1) | Brightness | 1 |  |
| (R2) | Reduce dullness | 2 |  |
| (R3) | Radiance | 3 |  |
| (R4) | Evened skin tone | 4 |  |
| (R5) | Smooth/Fine texture | 5 |  |
| (R6) | Relief of sensitivity | 6 |  |
| (R7) | Hydrated | 7 |  |
| (R8) | Oil-water balance | 8 |  |
|  | Others, please specify: __________ | 98 |  |

| O12 | Which of the following effects do you believe can be achieved through long-term (more than one week) anti-oxidation? [Multiple choice] | Code | Skip |
| --- | --- | --- | --- |
| (R1) | Smooth/Fine texture | 1 |  |
| (R2) | Fair skin | 2 |  |
| (R3) | Reduce yellowness | 3 |  |
| (R4) | Firmness | 4 |  |
| (R5) | Plump | 5 |  |
| (R6) | Tighten pores | 6 |  |
| (R7) | Acne marks/dark spots corrected | 7 |  |
| (R8) | Less sensitivity | 8 |  |
| (R9) | Oil-water balance | 9 |  |
| (R10) | Anti-wrinkles | 10 |  |
|  | Others, please specify: __________ | 98 |  |

**O2. Oxidation: category relevance**

| O2 | Which skincare benefit categories do you associate with anti-oxidation? [Multiple choice] | Code | Skip |
| --- | --- | --- | --- |
| (R1) | Skin and spot lightening | 1 |  |
| (R2) | Sunscreen | 2 |  |
| (R3) | Anti-acne | 3 |  |
| (R4) | Nourishing | 4 |  |
| (R5) | Repairing | 5 |  |
| (R6) | Anti-wrinkle | 6 |  |
| (R7) | Firming | 7 |  |
| (R8) | Soothing | 8 |  |
| (R9) | Oil control | 9 |  |
| (R10) | Exfoliation | 10 |  |
| (R11) | Hydration | 11 |  |
| (R12) | Cleansing | 12 |  |
|  | Others, please specify: __________ | 98 |  |
|  | None of the above | 99 |  |

**O3. Oxidation: expected time**

| O31 | How soon do you expect to see the short-term anti-oxidation effects you selected (insert O11 options) after using a skincare product? [Single choice] | Code | Skip |
| --- | --- | --- | --- |
|  | Immediately | 1 |  |
|  | Within half a day | 2 |  |
|  | Within 1 day | 3 |  |
|  | Within 1 week | 5 |  |

| O32 | How soon do you expect to see the long-term anti-oxidation effects you selected (insert O12 options) after using a skincare product? [Single choice] | Code | Skip |
| --- | --- | --- | --- |
|  | Within 2 weeks | 1 |  |
|  | Within 1 month | 2 |  |
|  | 1–3 months | 3 |  |
|  | 3–6 months | 4 |  |
|  | 6–12 months | 5 |  |
|  | More than 1 year | 6 |  |

**O4-O6 ask only if H2 = 2**

**O4. Oxidation: product usage**

| O4 | Which skincare products do you use to address oxidation? Please write at least one brand and product name.  Brand 1: __________ Product name 1: __________  Brand 2: __________ Product name 2: __________  Brand 3: __________ Product name 3: __________ |
| --- | --- |
|  |  |

**O5. Oxidation: satisfaction**

| O5 | Overall, how satisfied are you with the products you use for oxidation? Rate 1–5, 5=very satisfied, 1=very unsatisified. [Single choice] | Code | Skip |
| --- | --- | --- | --- |
|  | 5 | 5 |  |
|  | 4 | 4 |  |
|  | 3 | 3 |  |
|  | 2 | 2 |  |
|  | 1 | 1 |  |

**O6. Oxidation: low satisfaction reason**

| O6 | **Ask only if O5 = 1–3**  What are the reasons for your low satisfaction with the products you use for oxidation? [Open-ended]  _________________________________________________________________________________ |
| --- | --- |

**Skin Metabolism**

**M1. Skin Metabolism: expected effects**

| M1 | Which of the following effects do you believe can be achieved by improving skin metabolism? [Multiple choice] | Code | Skip |
| --- | --- | --- | --- |
| (R1) | Brightness | 1 |  |
| (R2) | Reduce dullness | 2 |  |
| (R3) | Refresh/mattified | 3 |  |
| (R4) | Radiance | 4 |  |
| (R5) | Rosy | 5 |  |
| (R6) | Hydrate | 6 |  |
| (R7) | Smooth/Fine texture | 7 |  |
| (R8) | Skincare products well absorbed | 9 |  |
| (R9) | Smooth/seamless makeup finish | 10 |  |
| (R10) | Depuffing | 11 |  |
| (R11) | Less sensitivity | 12 |  |
| (R12) | Relief of sensitivity | 13 |  |
| (R13) | Fortify skin barrier | 14 |  |
| (R14) | Acne marks/dark spots corrected | 15 |  |
|  | Others, please specify: __________ | 98 |  |

**M2. Skin Metabolism: category relevance**

| M2 | Which skincare benefit categories do you associate with improving skin metabolism? [Multiple choice] | Code | Skip |
| --- | --- | --- | --- |
| (R1) | Skin and spot lightening | 1 |  |
| (R2) | Sunscreen | 2 |  |
| (R3) | Anti-acne | 3 |  |
| (R4) | Nourishing | 4 |  |
| (R5) | Repairing | 5 |  |
| (R6) | Anti-wrinkle | 6 |  |
| (R7) | Firming | 7 |  |
| (R8) | Soothing | 8 |  |
| (R9) | Oil control | 9 |  |
| (R10) | Exfoliation | 10 |  |
| (R11) | Hydration | 11 |  |
| (R12) | Cleansing | 12 |  |
|  | Others, please specify: __________ | 98 |  |
|  | None of the above | 99 |  |

**M3. Skin Metabolism: expected time**

| M3 | How soon do you expect to see effects from improving skin metabolism after using a skincare product? [Single choice] | Code | Skip |
| --- | --- | --- | --- |
|  | Within 1 week | 1 |  |
|  | Within 2 weeks | 2 |  |
|  | Within 1 month | 3 |  |
|  | 1–3 months | 4 |  |
|  | 3–6 months | 5 |  |
|  | 6–12 months | 6 |  |
|  | More than 1 year | 7 |  |

**M4-M6 ask only if H2 = 4**

**M4. Skin Metabolism: product usage**

| M4 | Which skincare products do you use to improve skin metabolism? Please write at least one brand and product name.  Brand 1: __________ Product name 1: __________  Brand 2: __________ Product name 2: __________  Brand 3: __________ Product name 3: __________ |
| --- | --- |
|  |  |

**M5. Skin Metabolism: satisfaction**

| M5 | Overall, how satisfied are you with the products you use to improve skin metabolism? Rate 1–5, 5=very satisfied,1=very unsatisfied. [Single choice] | Code | Skip |
| --- | --- | --- | --- |
|  | 5 | 5 |  |
|  | 4 | 4 |  |
|  | 3 | 3 |  |
|  | 2 | 2 |  |
|  | 1 | 1 |  |

**M6. Skin Metabolism: low satisfaction reason**

| M6 | **Ask only if M5 = 1–3**  What are the reasons for your low satisfaction with the products you use to improve skin metabolism? [Open-ended]  _________________________________________________________________________________ |
| --- | --- |

**Glycation**

**G1. Glycation: expected effects**

| G1 | Which of the following effects do you believe can be achieved through anti-glycation? [Multiple choice] | Code | Skip |
| --- | --- | --- | --- |
| (R1) | Brightness | 1 |  |
| (R2) | Reduce dullness | 2 |  |
| (R3) | Radiance | 3 |  |
| (R4) | Evened skin tone | 4 |  |
| (R5) | Smooth/Fine texture | 5 |  |
| (R6) | Fair skin | 6 |  |
| (R7) | Reduce yellowness | 7 |  |
| (R8) | Firmness | 8 |  |
| (R9) | Anti-wrinkles | 9 |  |
| (R10) | Plump | 10 |  |
| (R11) | Tighten pores | 11 |  |
| (R12) | Acne marks/dark spots corrected | 12 |  |
| (R13) | Free from acne and blackheads | 13 |  |
| (R14) | Less sensitivity | 14 |  |
| (R15) | Relief of sensitivity | 15 |  |
|  | Others, please specify: __________ | 98 |  |

**G2. Glycation: category relevance**

| G2 | Which skincare benefit categories do you associate with anti-glycation? [Multiple choice] | Code | Skip |
| --- | --- | --- | --- |
| (R1) | Skin and spot lightening | 1 |  |
| (R2) | Sunscreen | 2 |  |
| (R3) | Anti-acne | 3 |  |
| (R4) | Nourishing | 4 |  |
| (R5) | Repairing | 5 |  |
| (R6) | Anti-wrinkle | 6 |  |
| (R7) | Firming | 7 |  |
| (R8) | Soothing | 8 |  |
| (R9) | Oil control | 9 |  |
| (R10) | Exfoliation | 10 |  |
| (R11) | Hydration | 11 |  |
| (R12) | Cleansing | 12 |  |
|  | Others, please specify: __________ | 98 |  |
|  | None of the above | 99 |  |

**G3. Glycation: expected time**

| G3 | How soon do you expect to see anti-glycation effects after using a skincare product? [Single choice] | Code | Skip |
| --- | --- | --- | --- |
|  | Within 1 week | 1 |  |
|  | Within 2 weeks | 2 |  |
|  | Within 1 month | 3 |  |
|  | 1–3 months | 4 |  |
|  | 3–6 months | 5 |  |
|  | 6–12 months | 6 |  |
|  | More than 1 year | 7 |  |

**程序员：G4-G6 ask only if H2 = 3**

**G4.** **Glycation: product usage**

| G4 | Which skincare products do you use to address glycation? Please write at least one brand and product name.  Brand 1: __________  Product name 1: __________  Brand 2: __________  Product name 2: __________  Brand 3: __________  Product name 3: __________ |
| --- | --- |
|  |  |

**G5. Glycation: satisfaction**

| G5 | Overall, how satisfied are you with the products you use for glycation? Rate 1–5, 5=very satisfied, 1=very unsatisfied. [Single choice] | Code | Skip |
| --- | --- | --- | --- |
|  | 5 | 5 |  |
|  | 4 | 4 |  |
|  | 3 | 3 |  |
|  | 2 | 2 |  |
|  | 1 | 1 |  |

**G6. Glycation: low satisfaction reason**

| G6 | **Ask only if G5=1/2/3**  What are the reasons for your low satisfaction with the products you use for glycation? [Open-ended]  _________________________________________________________________________________ |
| --- | --- |

**Exfoliation**

**EA1. Exfoliation: expected effects**

| EA1 | | Which of the following effects do you believe can be achieved through exfoliation? [Multiple choice] | | Code | Skip |
| --- | --- | --- | --- | --- | --- |
| (R1) |  | | Tighten pores | 1 |  |
| (R2) |  | | Remove blackheads | 3 |  |
| (R3) |  | | Refresh/mattified | 5 |  |
| (R4) |  | | Oily-free | 6 |  |
| (R5) |  | | Skincare products well absorbed | 7 |  |
| (R6) |  | | Smooth/seamless makeup finish | 8 |  |
| (R7) |  | | Comfort | 9 |  |
| (R8) |  | | Skin breathability | 10 |  |
| (R9) |  | | Reduce roughness | 11 |  |
| (R10) |  | | Smoother skin | 12 |  |
| (R11) |  | | Radiance/brightness | 13 |  |
| (R12) |  | | Reduce dullness | 14 |  |
| (R13) |  | | Skin is renewed/regenerated/Resurface the skin | 15 |  |
| (R14) |  | | Improve/refine skin texture | 16 |  |
|  |  | | Others, please specify: __________ | 98 |  |

**EA2. Exfoliation: product categories**

| EA2 | Which of the following product types do you associate with exfoliation? [Multiple choice] | Code | Skip |
| --- | --- | --- | --- |
|  | Cleansing product | 1 |  |
|  | Lotion/Toner | 2 |  |
|  | Acid serum | 3 |  |
|  | Cleansing mask | 11 |  |
|  | Sheet mask | 12 |  |
|  | Makeup remover | 13 |  |
|  | Facial scrub | 14 |  |
|  | Others, please specify: __________ | 98 |  |
|  | None of the above | 99 |  |

**End of the questionnaire**

**Supplementary Figure 3: A summary of the demographics of the women who took part in the qualitative focus group discussions (n=24 participants)**


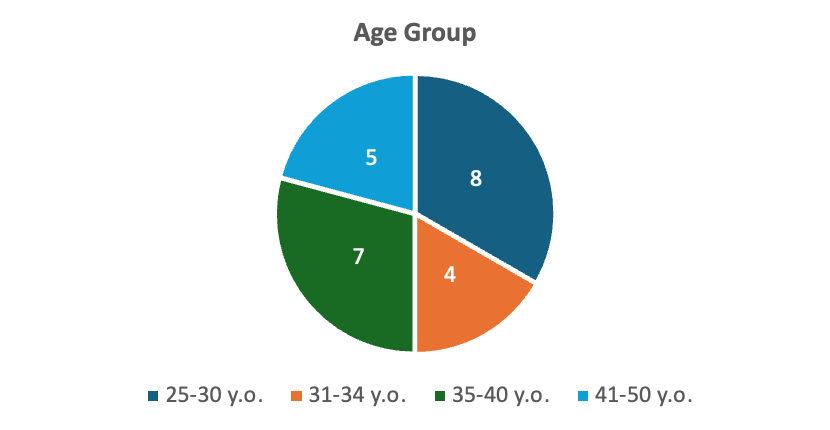


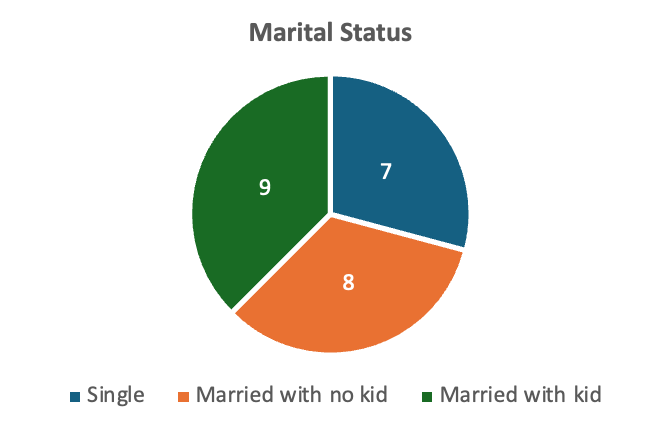


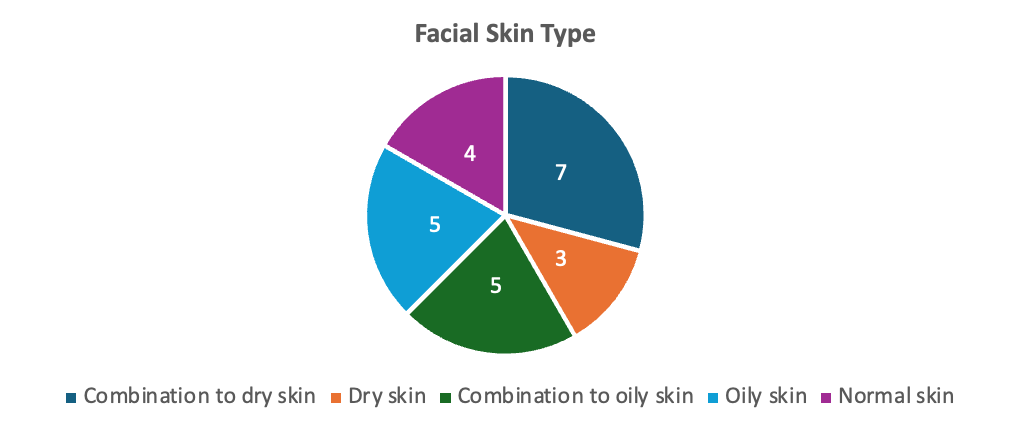


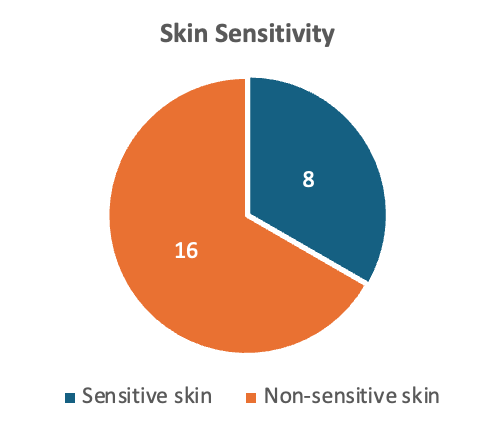


**Supplementary Figure 2 (continued): A summary of the demographics of the women who took part in the qualitative focus group discussions (n=24 participants)**


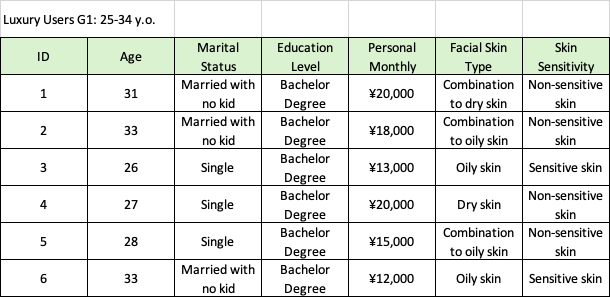


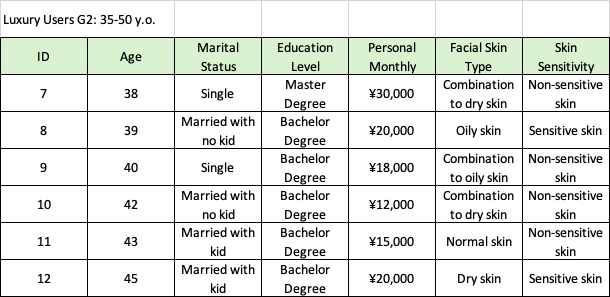


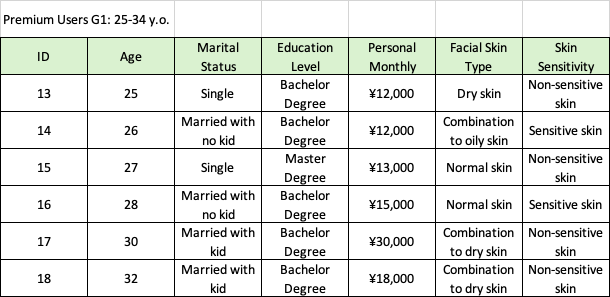


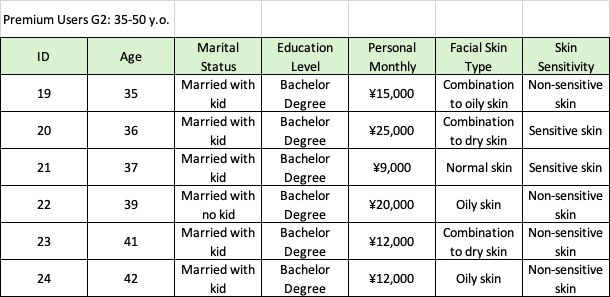

Supplement: Supplementary file 1 — Figure S1: Guide used by moderators for qualitative focus group discussions. Figure S2: Online questionnaire. Figure S3: A summary of the demographics of the women who took part in the qualitative focus group discussions (n = 24 participants). [file JOCD-24-e70623-s001.docx]
